# Towards next generation maggot debridement therapy: transgenic *Lucilia sericata* larvae that produce and secrete a human growth factor

**DOI:** 10.1186/s12896-016-0263-z

**Published:** 2016-03-22

**Authors:** Rebecca J. Linger, Esther J. Belikoff, Ying Yan, Fang Li, Holly A. Wantuch, Helen L. Fitzsimons, Maxwell J. Scott

**Affiliations:** Department of Entomology, North Carolina State University, Campus Box 7613, Raleigh, NC 27695-7613 USA; Institute of Fundamental Sciences, Massey University, Palmerston North, New Zealand

**Keywords:** Maggot debridement therapy (MDT), Platelet-derived growth factor (PDGF), Excretions/secretions (ES), Growth factor treatment, Diabetic foot ulcer, Tetracycline transactivator, *Lucilia sericata*, Wound healing

## Abstract

**Background:**

Diabetes and its concurrent complications impact a significant proportion of the population of the US and create a large financial burden on the American health care system. FDA-approved maggot debridement therapy (MDT), the application of sterile laboratory-reared *Lucilia sericata* (green bottle fly) larvae to wounds, is a cost-effective and successful treatment for diabetic foot ulcers and other medical conditions. Human platelet derived growth factor-BB (PDGF-BB) is a secreted dimeric peptide growth factor that binds the PDGF receptor. PDGF-BB stimulates cell proliferation and survival, promotes wound healing, and has been investigated as a possible topical treatment for non-healing wounds. Genetic engineering has allowed for expression and secretion of human growth factors and other proteins in transgenic insects. Here, we present a novel concept in MDT technology that combines the established benefits of MDT with the power of genetic engineering to promote healing. The focus of this study is to create and characterize strains of transgenic *L. sericata* that express and secrete PDGF-BB at detectable levels in adult hemolymph, whole larval lysate, and maggot excretions/ secretions (ES), with potential for clinical utility in wound healing.

**Results:**

We have engineered and confirmed transgene insertion in several strains of *L. sericata* that express human PDGF-BB. Using a heat-inducible promoter to control the *pdgf-b* gene, *pdgf-b* mRNA was detected via semi-quantitative PCR upon heat shock. PDGF-BB protein was also detectable in larval lysates and adult hemolymph but not larval ES. An alternative, tetracycline-repressible *pdgf-b* system mediated expression of *pdgf-b* mRNA when maggots were raised on diet that lacked tetracycline. Further, PDGF-BB protein was readily detected in whole larval lysate as well as larval ES.

**Conclusions:**

Here we show robust, inducible expression and production of human PDGF-BB protein from two conditional expression systems in transgenic *L. sericata* larvae. The tetracycline-repressible system appears to be the most promising as PDGF-BB protein was detectable in larval ES following induction. Our system could potentially be used to deliver a variety of growth factors and anti-microbial peptides to the wound environment with the aim of enhancing wound healing, thereby improving patient outcome in a cost-effective manner.

## Background

Diabetes is a global health care issue. Three hundred-eighty-two million people were reported to have diabetes in 2013 (http://www.diabetesatlas.org). Additionally, the cost of diabetic foot ulcers to the American health care system was estimated to be $9–13 billion in addition to care for diabetes in 2013 [1]. Maggot debridement therapy (MDT) is a cost-effective, FDA-approved treatment for diabetic foot ulcers [2, 3]. MDT commonly involves the application of sterile *Lucilia sericata* larvae to a non-healing wound to promote healing and decrease infection. MDT has been applied successfully in more than 20 additional medical conditions [4, 5].

MDT promotes healing in part through digestion and mechanical removal of necrotic tissue. Debridement is a critical component of effective wound healing [4, 6]. Enzyme application and mechanical debridement have been studied in clinical trials, but challenges such as expense and potential damage to healthy tissue stunt the large-scale effectiveness of these treatment options [7]. In contrast, larvae leave behind healthy tissue. Larvae have been shown to ingest fluorescent bacteria in vitro [8] as well as raise the pH of the wound environment via excretions and secretions (ES), which results in inhibition of bacterial growth [9]. Most of the in vitro studies found that ES was more effective at inhibiting growth of gram positive than gram negative bacteria [10]. Further, in one small vivo study, sterile maggots were found to be more effective at inhibiting growth of gram positive bacteria in infected wounds [11]. Specific factors and fractions have been identified within ES that exhibit antibacterial activity in vitro [12]. For example, the insect defensin homologue lucifensin was detected in the gut and salivary glands of *L. sericata* larvae and identified in wound washings from MDT patients [13]. Lucifensin exhibited antibacterial activity against a panel of Gram positive bacteria [13, 14]. Some data suggests expression and secretion of antibacterial factors by larvae is not constitutive, but induced by the wound environment [15–17]. The antibacterial mechanisms of MDT are free from the limitations of antibiotic resistance frequently seen in the clinic. Indeed, maggot debridement therapy has been shown to be effective in treatment of MRSA in vitro as well as in clinical case studies [18].

It is clear from these studies that larvae significantly alter the wound environment during MDT. Maggot ES may also alter the local inflammatory response. For example, *L. sericata* ES modulate neutrophil migration and adhesion and alter expression of pattern recognition receptor levels [19]. ES also increased the secretion of anti-inflammatory cytokine IL-10, while inhibiting secretion of pro-inflammatory cytokines TNF-alpha and IL-12p40 [20].

In addition to the impact of MDT on the immune response in the wound, MDT also promotes wound healing through formation of granulation tissue [21, 22]. This could be a consequence of the physical action of the maggots in the wound, removal of dead tissue, change of wound pH and microbial killing [10]. In addition, there is some evidence that maggot ES could stimulate growth of human cells in the wound. ES was shown to stimulate fibroblast proliferation in culture [23] and hepatocyte growth factor (HGF) synthesis in 3T3 cells [24]. Further, increased HGF levels were measured in femoral vein blood of patient during MDT [24]. However, there is no evidence from randomized clinical trials that MDT shortens wound healing times [25]. This may reflect a limitation of the design of the trials [10] but highlights the need for further studies on the promotion of wound healing by maggots.

Studies have shown decreased concentrations of growth factors, including several isoforms of platelet-derived growth factor (PDGF), in chronic wounds when compared to acute wounds [26, 27]. This evidence precipitated investigation into topical recombinant growth factor treatment as a means to promote healing in chronic wounds. Several growth factors were investigated, however molecular stability limited their success despite the use of gels, micropheres, and other conjugates. Nevertheless, after achieving some pre-clinical and clinical success, human PDGF-BB became the first FDA-approved recombinant cytokine growth factor [28].

The role of PDGF in wound healing is well established [29]. PDGF is a cationic hetero or homo-dimer consisting of a combination of alpha and beta subunit chains containing multiple intra- and inter-chain disulfide bonds. The subunits are produced in a pro form by endothelial cells, fibroblasts, immune system cells, and others [23]. The pro subunits dimerize in the endoplasmic reticulum into homo or heterodimeric combinations. The pro dimers are further processed via N-terminal modification, remodeling, and cleavage to a mature dimer form [30]. The mature dimers are secreted, where they interact with the extra-cellular matrix (ECM) and cell surface PDGF receptors. Via activation of the PDGF receptor and subsequently PI3 kinase and mitogen-activated protein kinase (MAPK), PDGF stimulates cell survival, fibroblast proliferation and chemotaxis, actin reorganization, and production and secretion of other growth factors, ECM constituents, and metalloproteases [29]. Because of the extensive role of PDGF in wound healing, clinical trials have been done investigating the utility of a topical gel (Becaplermin) containing recombinant human PDGF-BB produced in *Escherichia coli* [31]. Immunostaining of wounds treated with PDGF-BB showed increased fibroblasts, increased collagen fibril formation, and healing (as measured by decreased wound size) [32]. In one study, ulcer surface area and time to complete healing were both reduced significantly in patients receiving topical PDGF-BB along with standard wound care, however, the authors purposefully selected large, severe ulcers for inclusion in the study [33]. Similarly, other trials reported increased healing and/or reduced time to wound closure [34–41]. However, some trials did not find that topical PDGF treatment significantly improved wound healing [42–44]. The mixed outcomes could reflect the complexity of the wound healing process that involves mutiple factors, which supports the need for a therapy that combines multiple mechanisms to promote wound healing.

Here, we present a novel concept in MDT technology that combines the established benefits of MDT with the potential power of engineered maggots to promote healing. Genetically modified larvae engineered to secrete selected human growth factors or antibacterial peptides effective against Gram positive and Gram negative bacteria could have the potential to synergistically improve wound healing and result in shorter hospital stays. Given the low cost of rearing maggots, the technology is likely to be cost-effective compared to dressing gels containing recombinant proteins. The objective of this study is to determine if *L. sericata* can be engineered to conditionally express and secrete human PDGF-BB. PDGF-BB was selected because PDGF-BB made in *E. coli* is active and has been approved for use in wound treatment. However, we view this study as proof-of-principle for the future development of engineered *L. sericata* strains that express a variety of growth factors and antimicrobial peptides.

## Results

### Heat inducible expression of *pdgf-b* RNA in transgenic *L. sericata*

**Fig. 1:**
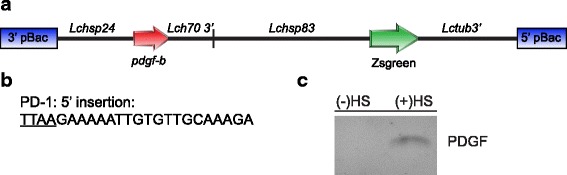
Heat inducible expression of *pdgf-b* mRNA in transgenic PD-1 *L. sericata*. **a** Schematic of heat-inducible *pdgf-b* gene construct in a *piggyBac* transformation vector with a ZsGreen marker gene. **b** Genomic DNA sequence adjacent to the 5' pBac end in the PD-1 transgenic line. The TTAA insertion site is underlined. **c** RT-PCR amplification of *pdgf-b* on total RNA obtained from first instar PD-1 larvae that had been given a heat shock (+HS) or no heat shock (-HS)

A heat-inducible system was chosen for *pdgf-b* expression as it provides several advantages. If fitness costs were observed, flies could be reared in non-permissive conditions in order to obtain sufficient numbers of larvae. Subsequently, during clinical application of larvae, the wound temperature would generate the permissive condition and induce *pdgf-b* expression. We previously showed that the *Lucilia cuprina hsp24* (*Lchsp24*) gene is strongly induced by heat shock in first and third instar larvae [45]. Further, several putative heat shock factor binding sites were identified within 500 bp upstream of the transcription start site. Thus the *Lchsp24* promoter was selected to make the heat inducible *pdgf-b* gene construct, pB[*Lchsp24*-*pdgf-B*] (Fig. 1a). The *Lchsp24* gene fragment contained 1016 bp upstream of the start of transcription and 180 bp of the 5' UTR. The translation start codon was not included in the fragment. A gene fragment encoding the mature active form of *pdgf-b* gene was synthesized with a codon usage optimal for expression in *Lucilia*. To facilitate secretion from *L. sericata* larvae, an amino terminal predicted signal peptide was included in the synthesized *pdgf-b* gene. The signal peptide was identified in a venom peptide that is expressed in *L. sericata* larval salivary glands [46]. For polyadenylation of *pdgf-b* transcripts, the gene construct contained the 3'UTR and 3' flanking DNA from a *Lchsp70* gene. The *Lchsp24*-*pdgf-b*-pA gene construct was inserted into a *piggyBac* transformation vector used previously [47] (Fig. 1a.). The vector contains a ZsGreen marker gene under the control of a strong constitutive promoter (*Lchsp83*) for identification of transgenic larvae.

*L. sericata* CA06 embryos were injected with pB[*Lchsp24*-*pdgf*-B] DNA and a *piggyBac* helper DNA/RNA mixture [47, 48]. Four transgenic lines were obtained from 18 G_0_ adults that developed from injected embryos. Two lines showed the predicted Mendelian inheritance for a single transgene (e.g. crossing heterozygotes with CA06 gave 50 % fluorescent offspring). Molecular analysis (inverse PCR) also indicated that these two lines (PD1 and PD2) carried a single copy of the transgene. However, only the PD1 line was homozygous viable and fertile and so this line was selected for further analysis. The nucleotide sequence adjacent to the the transgene was determined by inverse PCR (Fig. 1b). The transgene had inserted into a TTAA site, which is typical for *piggyBac*-mediated transformation [49].

To determine if *pdgf-b* mRNA expression was inducible by heat shock at 37 °C, RT-PCR was performed on RNA isolated from PD-1 homozygous first instar larvae. A DNA fragment of the correct size was detected from the heat-treated (37 °C for 30 min) but not control larvae (Fig. 1c). This suggests that the PD-1 line provides a heat inducible system for *pdgf-b* expression.

### Detection of PDGF-BB protein in PD-1 larval lysate

**Fig. 2:**
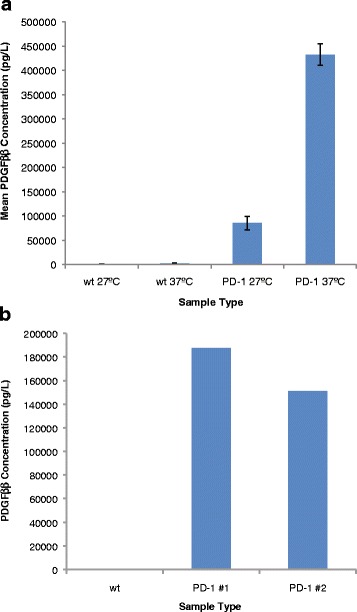
PDGF-BB protein is inducible in transgenic PD-1 *L. sericata* lysate and hemolymph. **a** Mean PDGF-BB concentration in wild type (wt) and PD-1 whole larval lysate under control and heat shock conditions. **b** PDGF-BB concentration in pooled wt or PD-1 adult hemolymph samples after heat shock. Data from two replicate experiments are shown

We next sought to determine whether or not the human PDGF-BB protein was detectable in lysates of transgenic larvae. A commercial ELISA kit was chosen due to its high sensitivity (as low as 15 pg/mL, according to the manufacturer). Further, ELISA assays detect native protein, while western blots detect linearized protein, allowing detection via antibodies against discontinuous as well as continuous epitopes, thereby further increasing likelihood of detection. For each assay, a positive control his-tagged recombinant human PDGF-BB was included, and yielded a positive signal within the kit standard range (data not shown). CA06 control (wt) and PD-1 larvae were subjected to a 3 h at 37 °C heat shock, then snap frozen. Lysates were normalized for total protein concentration and each sample was assayed in triplicate ELISA wells. PDGF-BB was undetectable in control CA06 lysate (Fig. 2a). While low basal levels of protein were detected in PD-1 larval lysate at the control temperature (27 °C), the PDGF-BB protein concentration increased 5–fold with heat shock treatment (Fig. 2a). For any future clinical application it is important that the PDGF-BB protein is secreted from *Lucilia* cells and is present in larval ES. Thus we next heat-shocked larvae in ES collection buffer and collected the ES. However, PDGF-BB was not detectable in larval secretions (data not shown). Total protein concentration was approximtely 10-fold lower in ES samples compared to whole larval lysates. As a result, the amount of total ES protein loaded per ELISA well was one tenth to one half that of lysates. It is possible that PDGF-BB was present in the ES samples but below the level of detection of the assay. We next collected protein from adult hemolymph, reasoning that the protein concentration would be higher than larval ES. PDGF-BB was detected in hemolymph isolated from adult PD-1 flies after heat shock, albeit at a lower concentration than lysates (Fig. 2b.) These data suggest that the transgenic expression system is functional, and that PDGF-BB is secreted into the hemolymph from cells in which it is expressed.

### *pdgf-b* RNA expression in transgenic *L. sericata* regulated by the tetracycline transactivator (tTA)

**Fig. 3:**
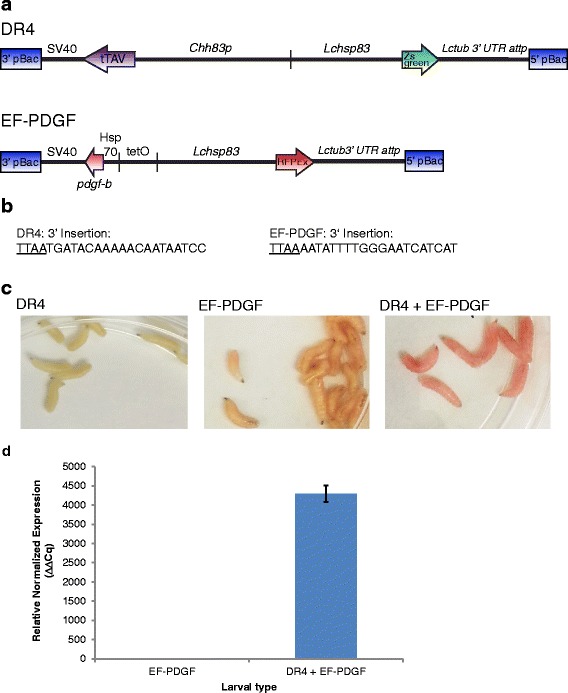
tTA-mediated *pdgf-b* expression in transgenic *L. sericata*. **a** Schematic of the DR4 tTA driver and EF-PDGF tTA-regulated effector gene constructs in *piggyBac* transformation vectors. **b** Genomic DNA sequence adjacent to 3' pBac for each strain. **c** DR4#14, EF-PDGF#11, and DR4#14 + EF-PDGF#11 larvae under white light. **d** Relative expression of *pdgf-b* mRNA in control effector alone and tTA-driver plus effector larvae. qRT-PCR analysis was performed on RNA isolated from whole larvae and normalized to the *28 s rRNA* reference gene

The above analysis suggested that higher levels of PDGF-BB expression were needed to produce detectable levels in larval ES. We had also previously shown that the “tet-OFF” system can lead to very high levels of gene expression in transgenic *Lucilia* [48]. The tet-OFF system is comprised of a tTA “Driver” construct and a tTA-responsive “Effector” construct (Fig. 3a). The *L. cuprina hsp83* promoter is a strong constitutive promoter in transgenic *L. sericata* [47]. Indeed, *L. sericata* larvae expressing DsRedex2 and ZsGreen under the control of this promoter appear light pink and greenish yellow respectively under white light (Fig. 3c). We reasoned that if the strong *hsp83* promoter was used to drive tTA expression, this would lead to very high levels of the effector, which in this study would be *pdgf-b*. Since the *L. cuprina hsp83* promoter was used for the marker gene, we isolated the *hsp83* promoter from a related blowfly, *Cochliomyia hominivorax* (*Chhsp83*). The start of transcription of the *Chhsp83* gene was determined by 5' RACE, using oligonucleotide primers based on a previously identified transcript [50]. The exon-intron arrangement was determined by PCR with genomic DNA template and primers based on the transcript. As for the *Lchsp83* gene [45], the *Chhsp83* gene contains one intron with the translation start codon at the beginning of the second exon. A PCR-based genome walking approach was used to obtain the nucleotide sequence of genomic DNA upstream from the start of transcription. The DR4 driver construct contains a 3 kb fragment from the *Chhsp83* gene upstream of the tTA coding region, followed by the SV40 polyadenylation sequence. The *Chhsp83* fragment includes 2225 bp of upstream flanking DNA, the 200 bp 5' UTR and the 586 bp first intron. The tTA translation start codon follows the *Chhsp83* intron. The *Chhsp83-tTA* gene cassette was cloned in a *piggyBac* transformation vector with a *Lchsp83-ZsGreen* marker gene. The EF-PDGF effector construct includes the *tetO*_*21*_*-Lchsp70* enhancer-promoter upstream of the *pdgf-b* coding region and SV40 polyadenylation sequence. The *tetO*_*21*_*-Lchsp70* enhancer-promoter was used previously to achieve high levels of tTA gene expression in *L. cuprina* (autoregulated system) [48]. The *pdgf-b* sequence with an N-terminal signal peptide was the same as used above in the heat inducible system. The *tetO-pdgf-b* gene cassette was cloned into a *piggyBac* transformation vector with a *Lchsp83-DsRedex2* marker gene.

Transgenic DR4 and EF-PDGF lines were obtained by *piggyBac*-mediated transformation. For DR4, 9 lines were initially obtained from 137 G_0_. However, the lines were weak and difficult to maintain. This is mostly likely because high levels of tTA protein are toxic to *L. cuprina* [48]. Consequently, only one line, DR4#14, was maintained and this was propagated as a mixture of heterozygotes and homozygotes. DR4 had been injected into *L. cuprina* embryos as part of the effort to make male-only lines [51]. For this study, it was necessary to introgress the DR4 transgene into a *L. sericata* genetic background. This was done by crossing *L. cuprina* DR4#14 males with MDLA *L. sericata* females. The offspring were then backcrossed for two generations with *L. sericata* females. The *L. sericata* EF-PDGF#11 line was obtained from 32 G_0_ and bred to homozygosity. The nucleotide sequence adjacent to the transgene insertion site was determined by inverse PCR (Fig. 3b).

To induce *pdgf-b* expression, the DR4#14 driver and and EF-PDGF#11 effector lines were crossed and the larval offspring collected. In the presence of the antibiotic tetracycline, tTA is bound by tetracycline and rendered ineffective. In the absence of tetracycline, however, tTA is able to bind to tetO in the effector construct and activate *pdgf-b* expression. Third instar larvae of the driver strain express ZsGreen marker, and appear yellow green in white light, while third instar larvae of the effector strain express the DsRedex2 marker and appear light pink. The progeny of this cross with both transgenes appear bright pink (Fig. 3c). This is most likely because tTA bound to tetO is also enhancing expression of the linked marker gene from the *Lchsp83* gene promoter. This was previously observed in larvae that overexpress tTA [48]. To confirm induction of *pdgf-b* transcript, RNA was isolated and quantitative RT-PCR was performed. *pdgf-b* transcript was readily detected in larvae that contain one copy of each of the DR4 and EF-PDGF transgenes. Control larvae (EF-PDGF only) had very low levels of *pdgf-b* mRNA expression (Fig. 3d). In larvae that had both transgenes there was a greater than 4000-fold increase in the level of *pdgf-b* RNA (Fig. 3d).

### PDGF-BB protein detection in whole larval lysate and ES from larvae heterozygous for the DR4 tTA driver and EF-PDGF effector

**Fig. 4:**
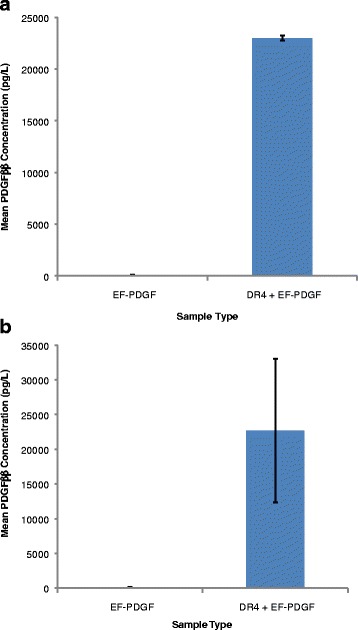
PDGF-BB protein is detectable in larval lysate and ES of larvae that carry both tTA driver (DR4) and tTA-regulated *pdgf-b* (EF-PDGF) transgenes. **a** Mean PDGF-BB concentration in control effector-alone and tTA-driver plus effector larvae. **b** Mean PDGF-BB concentration in effector-alone and tTA-driver plus effector larval ES

To determine if the larvae express and secrete PDGF-BB, ELISA assays were performed on ES samples collected from control EF-PDGF#11 larvae and larvae with both the DR4 and EF-PDGF transgenes. As with the heat inducible system, PDGF-BB was readily detected in whole larval lysate from larvae with driver and effector (Fig. 4a). When the more dilute ES samples were analyzed, total protein concentration was again much less than for lysates (30-fold). Therefore, the amount of total ES protein per ELISA well was one tenth to one half that of lysates . However, PDGF-BB was detected in ES samples from larvae that have both DR4 and EF-PDGF transgenes (Fig. 4b). Mean PDGF-BB concentration in ES was more variable between experiments than for whole larval lysates. Taken together, these data indicate that human PDGF-B is produced and secreted from third instar *L. sericata* larvae from a two-component transgene expression system.

## Discussion

Proteins of human and other origins have been expressed in insect cells in culture for decades [52]. More similar to our study, human protein has also been expressed in tissue of insects using transient viral-based [53] and transgenic systems [54]. For example, Medin and colleagues reported detection of recombinant human adenosine deaminase, a primarily non-secreted protein, in *Trichoplusia ni* larval lysate after injection of a baculovirus-mediated transgene [53]. Interest in expressing heterologous proteins in insect larvae has arisen from the desire for greater scale-up capabilities than is feasible using cells in culture. A more recent study demonstrated expression of a recombinant mouse anti-botulinum antibody fragment (Fab) in *Trichoplusia ni* larvae [55]. A secretory signal from *Bombyx mori* was utlilized to facilitate secretion of the protein product from larval cells, however, protein was purified from whole larvae. Lastly, secreted human proteins, including growth factors, have been expressed in *Bombyx mori* and purified from hemolymph as well as larval/pupal homogenate, and several of these proteins have proven functional in the veterinary clinic [56]. We show here for the first time that the translational and secretory mechanisms of the *L. sericata* larvae is capable of producing a human growth factor from our transgene expression systems, and that this protein is indeed detectable in hemolymph, larval lysate,and larval secretions. Further, we introduce the first human transgene expression in a larval host with potential for human clinical applications.

Two conditional systems were employed in this study, one regulated by temperature and the other by addition of tetracycline to the insect diet. The temperature-regulated system was chosen because *pdgf-b* expression would theoretically increase upon application of the larvae to patient. Although *pdgf-b* expression driven by the *Lchsp24* promoter was heat-inducible in whole larvae, we failed to detect PDGF-BB protein in maggot ES. Thus this conditional expression system would not be useful for any future clinical application. The *D. melanogaster hsp23* gene shows tissue-specificity and a cell-specific heat-shock response [57], therefore it’s possible that *Lchsp24* promoter has low activity in *Lucilia* larval tissues that excrete/secrete proteins found in ES. Nevertheless, our finding that the *Lchsp24* promoter is active and heat inducible could be useful for conditional expression of other proteins in blow flies. Further, PDGF-BB was detected in adult hemolymph after heat shock. This suggests that the pre-protein was likely correctly processed and secreted from *Lucilia* cells.

Using the two-component tet-OFF system, we have demonstrated that human PDGF-BB can be expressed in *L. sericata* larvae and detected in ES in an efficient inducible system. With this conditional system, larvae would be raised on diet without tetracycline to induce expression before wound application, thereby maximizing delivery of the secreted factor and healing potential. A strain could be potentially engineered with several different tetO-effector genes that would be co-regulated by tTA. For example, the effector genes would be other growth factors such as basic fibroblast growth factor [58] and/or antimicrobial peptides such as lucifensin [13] or Cecropin B [59]. The ES would be active against a broad spectrum of bacterial species as lucifensin is active against Gram positive bacteria [13] whereas Cecropin B is particularly active against Gram negative bacteria [59]. In the long term, clinicians might have at their disposal a panel of larvae expressing different factors for each individual wound based on the species of bacteria present in an infection, stage of wound healing, and level of inflammation.

The DR4 driver in our system may be further improved. The DR4 driver is not the best choice for building strains intended for clinical application, as high levels of tTA expression are toxic for *Lucilia*. A tTA driver employing a larval salivary gland-specific gene promoter would be advantageous [46, 60], as confining tTA expression to one tissue would likely have a lower fitness cost for the insect. Further, any effector protein induced by tTA would be secreted from the salivary gland. Alternatively, since larval excretions contribute to maggot ES, tTA could be expressed in the gut using a gut-specific promoter. The tet-OFF system could not be utilized in the clinic if tetracycline or a derivative was being used to treat an infected wound. If this proved to be problematic, the two-component GAL4-UAS system, widely used in *Drosophila* [61], could be considered for protein expression in *Lucilia*. With this system, it should be possible to achieve similar levels of protein expression as with the tet-OFF system, however expression would not be conditional. Lastly, with the identification of a *L. sericata* microbiome [62], a paratransgenesis approach [63] of utilizing a bacterial species to express a protein(s) of interest in the gut of *L. sericata* maggots could be considered.

As with any treatment modality, clinical application of genetically modified *L. sericata* would require regulatory approval. A potential challenge for the utility of modified MDT is patient attitude toward maggot application. However, several studies indicate that patients will accept MDT [64, 65]. Indeed, Steenvoorde et al., indicate that 89 % of patients surveyed would undergo MDT again and 94 % would recommend it to other patients [65]. Further, with the availability of polymer Biobags or pouches made of nylon or chiffon fabric [66], it may be possible to provide a liquid-permeable barrier when applying sterile genetically modified (GM) maggots to a wound, thereby making the treatment significantly more tolerable for opposing patients. It would also be anticipated that some patients would reject treatment with GM maggots given the public opposition to GM crops [67]. However, the GM maggots could be more acceptable, as the use of a fluorescent protein marker should facilitate thorough removal of maggots from a wound after treatment. The larvae could be readily visualized using goggles equipped with the appropriate filter sets [68].

Future studies are in order to further characterize the potential clinical utility of our system. Treatment of cultured fibroblasts with ES samples to measure MAPK activation, DNA synthesis, proliferation, and motility using an in vitro wound assay would confirm functionality of the secreted protein. Further, a rat wound healing model has been created [69] that would provide an excellent pre-clinical model.

## Conclusions

Here we show robust, inducible production of human *pdgf-b* RNA and PDGF-BB protein from two conditional expression systems in transgenic *L. sericata* larvae. With the heat-shock inducible system, PDGF-BB protein was detectable in hemolymph and whole larval lysate but not ES. The tTA-regulated system is more promising as PDGF-BB protein was detected in larval ES in addition to whole larval lysate from larvae raised on diet without tetracycline. Potentially, larvae could be engineered with several tTA regulated genes such that they secrete/excrete a variety of growth factors and/or antimicrobial factors with the aim of enhancing wound healing, thereby improving patient outcome. The International Diabetes Federation reports that 80 % of people with diabetes live in low or middle income countries (http://www.idf.org/diabetesatlas). Enhanced MDT may be a cost-effective solution for patients with less access to other treatment modalities.

## Methods

### Insect rearing and germline transformation

The CA06 and MD wild type strains of *L. sericata* were raised under similar conditions as described previously [48, 70]. To make the MDLA mixed strain, 16 MD males were mated with 30 CA06 virgin females, the offspring collected and reared to adults. For heat shock experiments, MDLA or *L. sericata* PD1 eggs were placed on MYEA (50 g whole egg powder, 25 g instant non-fat milk powder, 12.5 g inactivated dry yeast, 7.5 g agar, 500 mL deionized water) at 27 °C overnight. First instar larvae were transferred to a new MYEA dish then heat shocked at 37 °C or left at 27 °C control for 3 h. The larvae were collected and then washed with water using a filter, attached to an aspirator. Larvae were then snap frozen in liquid nitrogen and stored at -80 °C. Introgression of the DR4#14 line into a *L. sericata* genetic background was done by crossing 125 DR4#14 *L. cuprina* males with 80 MDLA virgin females. Male offspring were then crossed with MDLA virgin females for two generations. The male and female offspring allowed to breed freely to establish the line.

*piggyBac*-mediated germ-line transformation of *L. sericata* was as previously described [47, 48] using a mixture of *Lchsp83*-pBac DNA (200 μg/mL) and in vitro synthesized *piggyBac* RNA helper (300 μg/mL). Homozygous *Lucilia* individuals were selected at the wandering third instar larval stage based on fluorescence intensity and bred to create a stable line.

### Plasmid construction

To make the pB[*Lchsp24*-*pdgf*-B] construct, firstly the *Lchsp24* promoter [45] was amplified from *L. cuprina* genomic DNA with the primer pair 5'-GAGCTCCTCGAGTAGGGTGGGCAATTTTTTCTAATGCCCATTA-3' and 5'-GGATCCTGGATAGGCTTCACGGTCCAGTTCATCGAT-3' and the fragment inserted into pBAC2 vector [47]. The *Lchsp70* 3' UTR and 3' flanking DNA [45] were amplified from *L. cuprina* genomic DNA with the primer pair 5'-AAGCTTAGTCAATCTCAATTTCATTCC-3' and 5'-GCGGCCGCCTCGAGAATGATATATACAAGGA-3', and inserted downstream of the *Lchsp24* promoter. However, the *Lchsp24* promoter fragment retained the start codon, so this was removed by amplification of a portion of *Lchsp24* without the start codon with the primer pair 5'-ATTATCATTATCTACTAGTTCAGTTCTAGTTAC-3' and 5'-ATTATCGGATCCCTCTTTGGTTTTCTTAAA ACG -3', then digested with SpeI and BamHI(blunt) and inserted into SpeI and AgeI(blunt) of pB[Lchsp24-pl-Lchsp70], effectively replacing the promoter with an identical fragment minus the ATG codon. A DNA fragment was synthesized by Genscript that encoded the human PDGF-B protein and a 20 amino acid signal peptide (MKSFLLVLFAFLAVFAFVQA) from an 87 amino acid venom peptide that was expressed in *L. sericata* salivary glands [46] and detected in larval ES (P.H. Nibbering, personal communication). The nucleotide sequence was optimized for expression in *L. cuprina*. The *pdgf*-B Genscript plasmid was digested with flanking Asp718 (blunt) and AvrII and inserted into NheI (blunt) and AvrII of pBAC2[Lchsp24-Lchsp70]. A PspOMI and partial SpeI digest of pB[Lchsp83-ZsGreen-tub.2] [47] released a 3.3 kb Lchsp83-ZsGreen-tub.2 cassette which was inserted into PspOMI and SpeI of pBSII KS+. The resulting pBSII KS+[Lchsp83-ZsGreen-tub.2] plasmid was digested with NotI and PspOMI and inserted into NotI digested pB[*Lchsp24*- *pdgf*B -*Lchsp70*] to create the final *piggyBac* transformation vector, pB[*Lchsp24*-*pdgf-B*].

To construct DR4 and EF-PDGF, the general strategy was to first assemble the driver or effector gene cassette in the cloning vector pBluescript II KS (-) and then excise the gene cassette by digestion with XhoI and NotI and clone into the unique XhoI and PspOMI sites in the *piggyBac* transformation vectors pB[*Lchsp83*-*ZsGreen*] [47] or pB[*Lchsp83*-*DsRedex2*] [48]. To assemble the effector construct, a fragment encoding PDGF-B with N terminal signal peptide was amplified from pB[*Lchsp24*-*pdgf-B*] using the primer pair 5'-TTATCATGAAGTCGTTCTTGTTGGTGTTG-3' and 5'-TGAAAGCTTAGGTCACGGGACGGGCGGCAG-3', digested with BspHI and HindIII, and ligated to pBS-FL11 (Li et al. [48]) that had been digested with NcoI and HindIII. The effector gene cassette was then excised from the pBS-EF-PDGF plasmid and ligated with the pB[*Lchsp83*-*DsRedex2*] transformation vector.

For 5' RACE, total RNA was extracted from *C. hominivorax* embryos using TRI reagent (Sigma). polyA+ RNA was purified by oligo-dT chromatography (Sigma). 5' RACE-ready cDNA was prepared using the SMARTer RACE cDNA amplification kit (Takara) according to the manufacturers instructions. RACE-ready cDNAs were diluted in 20 μL TE buffer (Clontech), and then 2 μL was used for 5' RACE with the primer 5'-CAATTCACGCAAGAAAATCTCTTTGTTGGAATAGAAGGT-3'. “Genome walker” libraries were prepared from *C. hominivorax* genomic DNA using the Universal GenomeWalker kit (Takara) following the manufacturers instructions. PCR was then performed using the *Chhsp83* gene specific primers (GSP) 5'-GATCAACCACAATCTAATATATTATAACTTTTTTCACTTTTCAGTT-3' and 5'-TTGTCTTTTCGCTCGCTTGGAAACTCTCGATGTAT-3'. The *Chhsp83* gene promoter fragment for the DR4 construct was obtained by PCR amplification with genomic DNA template using the primers 5'-ATAGCGGCCGCTGTCATTACTAGAGTTTAAGTTATAACAATTGTAT-3' and 5'-ACGCTGCAGATCTGGAAATACAATAGGAAAAATAAAGTTAGCGAATT-3', then cloned into pGEM-T (Promega). The *Chhsp83* promoter fragment was excised and ligated with pBS-FL1 that had been digested with NotI and PstI, essentially replacing the *tetO*-*Dmhsp70* enhancer-promoter with the *Chhsp83* promoter. The *Chhsp83*-tTAv-SV40 gene cassette was then excised and ligated with the pB [*Lchsp83*-*ZsGreen*] transformation vector.

### Genomic DNA isolation and inverse PCR

Five to 6 frozen adults were ground to powder with a mortar and pestle under liquid nitrogen. Powder was dissolved in 4 mL fresh STE buffer (50 mM Tris-HCl, pH 7.5, 100 mM NaCl, 10 mM EDTA, pH 8). Two hundred μL 10 % SDS and 8 μL RNase A (Cat# R4642-250MG Sigma Aldrich St. Louis, Missouri) were added and samples were incubated at 56 °C. After 30 min, Proteinase K (Cat# P2308-100MG Sigma Aldrich) was added to 100 μg/mL and the sample was incubated overnight at 56 °C. Three mL phenol:chloroform:isoamyl alcohol [25:24:1] (Cat#P2069, Sigma) was added and samples were rotated 10 min at 12 RPM at 22 °C. Samples were then centrifuged 10 min at 1000 g at 4 °C. The aqueous layer was transferred to a new tube and the extraction was repeated. One tenth volume 3 M sodium acetate, pH 5.2 and 2 volumes cold 100 % ethanol were added and mixed. The samples were incubated at -20 °C for 1 h and centrifuged 30 min at 7200 g at 4 °C. The supernatant was removed from the pellet, which was washed with 1 mL cold 75 % ethanol. The pellet was air-dried 10 min before resuspension in 50–100 μL TE Buffer. To determine genomic sequence flanking the transgene insertion, inverse PCR was performed with MboI, TaqαI, and MspI-digested genomic DNA templates as described previously [48, 71].

### RT-PCR and qRT-PCR

RT-PCR was performed with cDNA template from total RNA isolated from larvae as described previously [72]. The *pdgf-b* primer pair used were PDGF-F (5-' ATG AAG TCG TTC TTG TTG GTG TTG TTC GCC TTC TTG GCC GTT-3') and PDGF-R (5'- CCG GAG TTT AAA CCC TAG GCG CGC CAT GAG CTC AAG CTT TCA TTA-3'). For RNA isolation for quantitative RT-PCR (qRT-PCR), 5-6 frozen larvae were homogenized in 500 μL of Trizol (Cat#15596026 Life Technologies/ Thermo Fisher Scientific Waltham, Massachusetts) in a 1 mL glass homogenizer that had been previously baked at 200 °C overnight. 100 μL of chloroform was added, and samples were shaken for 15 s and allowed to incubate at 22 °C for 15 min. Samples were centrifuged at 18,000 g for 15 min at 4 °C. The aqueous layer was mixed with an equal volume cold RNase-free 70 % ethanol, mixed, and loaded on a Qiagen RNeasy Mini Kit column (Cat#74104 Qiagen Venlo, Netherlands). The purification was performed according to the kit protocol, including the optional on-column DNase digest using the RNase-free DNase set (Cat#79254 Qiagen). A subsequent in-solution DNase digest was performed to eliminate residual DNA, followed by a second round of column purification. cDNA was synthesized from 3.5 μg of DNase treated RNA using Superscript III (Cat#18080-400 Invitrogen) according to manufacturer's instructions. Random hexamers were used as primers. Negative control reactions containing water instead of enzyme mix were performed to confirm the absence of DNA contamination.

PDGF qPCR primers were designed using Primer3: hPDGF F (5'- GAAATTGTGCGTAAAAAGCCCATTT-3') and hPDGF R (5'- AACAGTTTCACATTTACAGGCCAAA-3'). Primers were tested for efficiency by creating a dilution series of cDNA. Template was pipetted into quadruplicate wells of a 384 well optical plate (Cat#4309849 Applied Biosystems). Thermo Maxima SYBR Green/ Rox qPCR Master Mix 2X (Cat#K0221 Thermo Fisher Scientific Waltham, Massachusetts) was added to primers to create a master mix, which was dispensed into wells via a multi-channel pipet. The plate was sealed (Cat#4311971 Applied Biosystems), mixed, then centrifuged 1 min at RT at 1600 g. The qPCR run was performed on a BioRad CFX384 C1000 Touch Thermocycler (BioRad Hercules, CA) using the following program: 95 °C 10 min, [95 °C 15 s, 60 °C 60 s, 40x]. Data acquisition was performed on the anneal/ extension step. Primer efficiency was determined by plotting the log of the starting template dilution on the X-axis and the mean Cq of the quadruplicate replicates on the Y-axis. The slope of the best fit line was used in the following equation to calculate efficiency: [Efficiency = -1 + 10^(-1/slope)^]. Primers were accepted if efficiency was 90–105 % and re-designed if efficiency fell outside this range.

For measurement of relative transcript levels, cDNA templates were diluted 1:4 with nuclease-free water then pipetted into quadruplicate wells of a 384 well optical plate for each primer set, hPDGF and the 28 s rRNA reference gene. The 28S rRNA primer pair were Lc-28SF (5'-ACCACTGTTCACACGAAACCCTTC-3') and Lc-28SR (5'-ATCTCGGTTGGATTTTAAACTTTGAAA-3'). The qPCR protocol was performed as above. Analysis of delta delta Cq was performed using BioRad CFX Manager. Mean Cq value was found for each set of 4 replicate wells. The reference gene was utilized to calculate ΔCq. The EF-PDGF control was chosen as the calibrator sample and set to 1. The bar graph represents ΔΔCq (relative normalized expression), with error bars representing standard error of the mean for the replicate values.

### Protein analysis

With the PD1 line (*Lchsp24-pdgfb*), prior to hemolymph collection, adult flies were heat shocked at 37 °C for 2 h with access to water and allowed to recover at room temperature for 3 h. Flies were anesthetized by exposure to carbon dioxide and then one wing was removed. The hemolymph was squeezed into a capillary tube pre-filled with a small amount of cold hemolymph collection buffer (10 mM EDTA pH7 in 1X phosphate-buffered saline (PBS) + 1X Protease inhibitor cocktail (Cat# P2714, Sigma-Aldrich, St. Louis, MO) on ice. Samples were centrifuged 2 min at 10,000 g at 4 °C. Supernatant was transferred to a new tube. Multiple adults were pooled for one sample. Samples were stored at -80 °C.

For offspring of the cross between DR4 driver and EF-PDGF effector, third instar larvae were sorted by fluorescence and rinsed with water on a vacuum funnel with gentle suction. Larvae were briefly placed on a Kim Wipe to remove excess moisture before being placed in Eppendorf tubes and snap frozen in liquid nitrogen. For ES collection, larvae were processed as above and then placed into wells of a 12-well flat-bottomed polystyrene tissue culture plate. Six or 50 larvae were added per well. Two hundred μL (for 6 larvae) or 1 mL (for 50 larvae) of ES collection buffer (Ringer Solution [0.120 mM NaCl, 1.5 mM CaCl_2_, 5 mM KCl, pH 7.4, filter sterilized] + 1X Protease inhibitor cocktail) was added per well, and wells were sealed with an adhesive plate sealer. After 40 min, ES was removed and centrifuged at 21,000 g for 30 min at 4 °C. Supernatant was transferred to a new tube and stored at -80 °C, and debris pellet was discarded. Frozen larvae were lysed on ice in cold gentle lysis buffer [73] using 1 mL glass homogenizers. Following homogenization, lysates were centrifuged at 15,000 g 15 min at 4 °C. Supernatant was transferred to a new tube and stored at -80 °C, while debris pellet was discarded.

Total protein concentration was determined for lysates and hemolymph using the Pierce BCA Protein Assay Kit (Cat#23227, Thermo Fisher, Rockford, IL) and for ES using Quickstart Bradford 1X Dye Reagent and Quickstart Bovine Gamma Globulin Standard (Cat# 500-0205 and 500-0208, BioRad, Hercules, CA). The hPDGF-BB Quantikine ELISA kit was purchased from R&D Systems (Cat# DBB00) and assay was performed according to kit protocol. Lysate and ES samples were assayed in triplicate wells, containing 150 μg and 15–60 μg protein per well respectively. Eighty μg of total protein from each hemolymph sample was assayed per well. Recombinant His-tagged human PDGF-BB (Cat# ab73231, Abcam, Cambridge, MA) was assayed on each ELISA run and provided a positive, in-range control for the integrity of the kit. Sample concentrations were extrapolated or interpolated from a standard curve constructed from the log PDGF-BB concentration (pg/L) on the X-axis and the log mean optical density reading (OD) on the Y-axis. Error bars represent standard error of the mean for the replicate values.

### Ethics approval and consent to participate

“Not applicable” as this study did not involve any animal or human data or tissue.

### Consent for publication

“Not applicable”.

### Availability of data and material

The GenBank accession numbers for the plasmids made in this study are:

- pB[*Lchsp24*-*pdgf-B*]: KT897708
- DR4: KT897710
- EF-PDGF: KT897709

## Abbreviations

cDNA: Complementary DNA
*Ch*: *Cochliomyia hominivorax*
Cq: Quantification cycle
ECM: Extra-cellular matrix
EDTA: Ethylenediaminetetraacetic Acid
ELISA: Enzyme-linked immunosorbent assay
ES: Excretions/secretions
FDA: Food and Drug Administration
GM: Genetically modified
GSP: Gene specific primers
h: Hours
HGF: Hepatocyte growth factor
hPDGF: Human Platelet-Derived Growth Factor
Hsp: Heat shock protein
IL-#: Interleukin
kb: Kilobase
*Lc*: *Lucilia cuprina*
*Ls*: *Lucilia sericata*
MAPK: Mitogen-activated protein kinase
MDT: Maggot debridement therapy
mRNA: Messenger RNA
MRSA: Methicillin resistant *Staphylococcus aureus*
MYEA: Milk, yeast, egg, agar larval media
OD: Optical density
pBac: *piggyBac*
PBS: Phosphate-buffered saline
PCR: Polymerase chain reaction
PDGF-B: Platelet-Derived Growth Factor Beta
RACE: Rapid Amplification of cDNA Ends
rRNA: Ribosomal RNA
RT: Reverse transcriptase
s: Seconds
SDS: Sodium dodecyl sulfate
TE: Tris- EDTA
Tet: tetracycline
tetO: Tetracycline operator
TNF-alpha: Tumor necrosis factor alpha
tTA: Tetracycline transactivator
μg: Microgram
UTR: Untranslated region
